# Targeting Mutant Protein Kinase B with Lysine-Selective Salicylaldehyde Inhibitors and Zn^2^⁺ Chelation: A Novel Therapeutic Strategy

**DOI:** 10.1186/s43556-025-00278-3

**Published:** 2025-06-06

**Authors:** Runpeng Yu, Yiran Wu, Long Zhang

**Affiliations:** 1 Department of Radiation Oncologyand the, State Key Laboratory of Transvascular Implantation Devices , The Second Affiliated Hospital of Zhejiang University School of Medicine, Life Sciences Institute, Zhejiang University, Hangzhou, China; 2 The MOE Basic Research and Innovation Center for the Targeted Therapeutics of Solid Tumors, The First Affiliated Hospital, Jiangxi Medical College, Nanchang University, Nanchang, China; 3 Frontiers Medical Center, Tianfu Jincheng Laboratory, Chengdu, China

In a recent study published in *Nature*, Craven et al. introduced a novel, mutant-selective AKT inhibition strategy [1]. The study demonstrated that the most common mutation site in AKT1 (the serine/threonine-protein kinase Akt, also known as protein kinase B), E17K, can be effectively targeted using specially designed salicylaldehyde-based inhibitors, referred to as Compounds 1–3 in the study. Additionally, the researchers discovered the alterable AKT1 (E17K)-salicylaldehyde compound could be enhanced in stability by forming the ‘neo-Zn^2+^-chelate’, indicating the detailed molecular mechanism of the compounds.

AKT1 plays a vital role in cellular processes such as proliferation, survival, metabolism, and protein synthesis [2] (Fig. 1a). It is a key component in the PI3K-AKT-mTOR signaling pathway, which regulates critical cell processes. In this pathway, activation begins with PI3K producing PIP3, which recruits AKT1 to the plasma membrane. Phosphorylation of the AKT1 at Thr308&Ser473 residues then fully activates its kinase domain, triggering its downstream phosphorylation cascades. Mutations in AKT1 are strongly associated with the onset of several solid tumors, with the Glu17-to-Lys (E17K) mutation being the most prevalent. This mutation has been linked to the development of breast cancer [3], meningiomas [4], and endometrial carcinomas [5]. Although clinical AKT-targeting inhibitors have been developed for treating AKT1 mutation-related cancers, they often target multiple AKT variants (AKT1, AKT2, and AKT3) and cause side effects like hyperglycemia, limiting their clinical usefulness. Thus, a more precise AKT1(E17K)-specific chemical inhibitor with minimal side effects is needed.

**Fig. 1:**
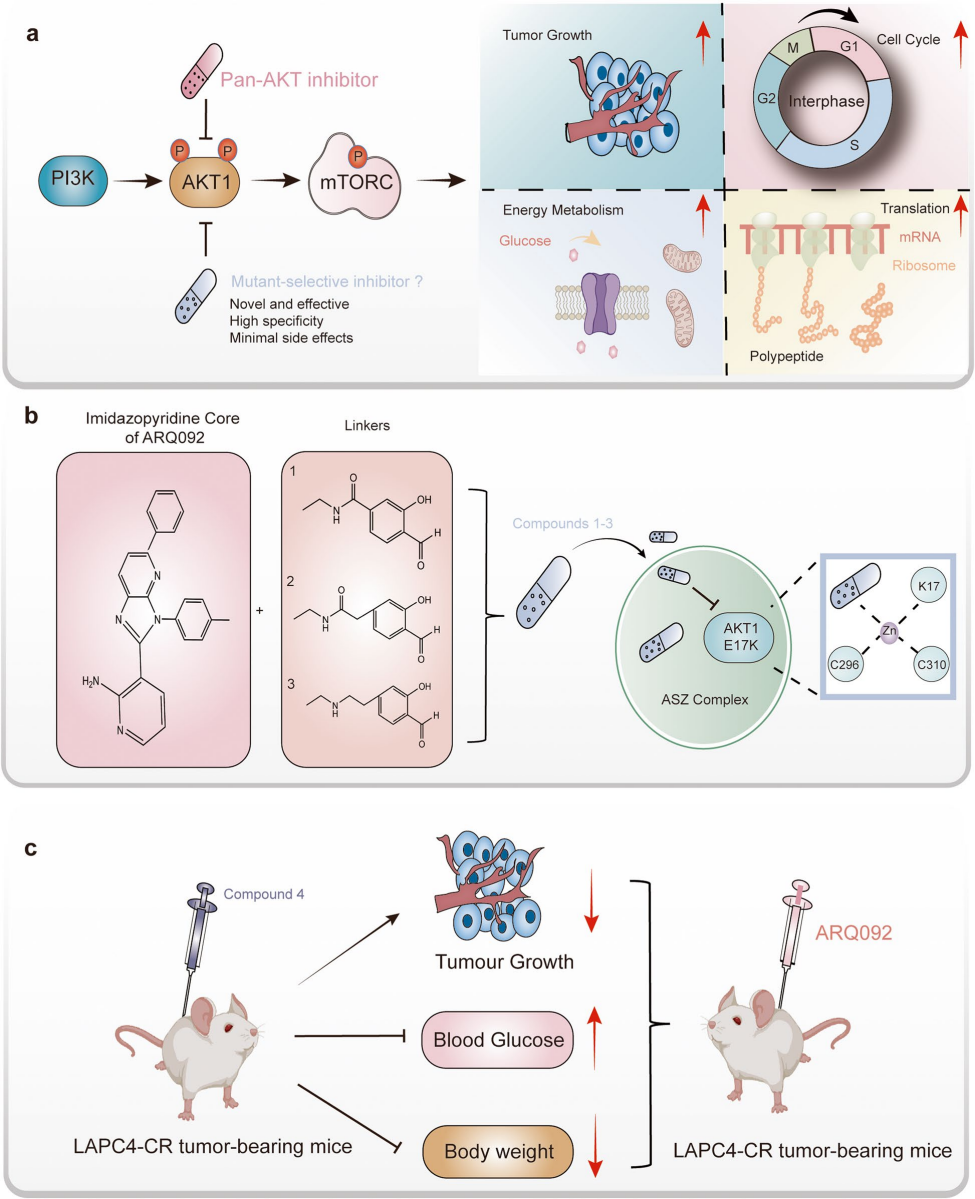
Overview of the Mechanism of Mutant-Selective AKT1 Targeting Therapy and the Therapeutic Effects of Synthetic Compounds on Cancer. **a** Cellular functions of the downstream AKT-mTOR signaling pathway. The AKT-mTOR pathway regulates several key cellular processes, including tumor growth, cell cycle progression, energy metabolism, and translation control. **b** Chemical structures of Compounds 1–3 and their mechanism of action against AKT1(E17K). Compounds 1–3 are based on the imidazopyridine core of ARQ092 and feature different linker structures. These compounds demonstrate enhanced targeting of the AKT1(E17K) mutant. The unique AKT1(E17K)-salicylaldehyde-Zn^2+^ complex (the ASZ complex in the article) stabilizes the binding affinity between the compounds and the mutant AKT1. **c** In vivo evaluation of mutant-selective compounds. Compound 4 effectively inhibits the growth of AKT1(E17K)-mutant tumors in vivo, with minimal side effects on blood glucose and body weight

To address this issue, Craven et al. utilized computational modeling to analyze how AKT1 interacts with the allosteric inhibitor ARQ092. They hypothesized that the chemical compounds which composed of ARQ092 with different linkers to a salicylaldehyde (Fig. 1b), might bind to Lys17, a key site in the E17K mutation. Building on this hypothesis, the authors synthesized three compounds, designated Compounds 1–3 (Fig. 1b). Notably, both in vitro mass spectrometry and differential scanning fluorimetry revealed that Compounds 1–3 covalently bound to AKT1(E17K) with greater selectivity than the pan-AKT inhibitor ARQ092. Furthermore, LC–MS/MS analysis also validated that the Lys17 was the primary site of the covalent binding. Taken together, these findings suggest that Compounds 1–3 could serve as potential chemical inhibitors targeting the mutated AKT1.

Next, the authors tried to uncover the covalent engagement of Compounds 1–3 in cells. They performed thermal shift assays in BEAS-2B cells that stably overexpressing AKT1(WT), AKT1(E17K), and AKT2 with flag tag, respectively. The results revealed that Compound 3 induced the thermal stability improvement of FLAG-AKT1(E17K) compared to ARQ092. For direct visualization, the authors synthesized a salicylaldehyde-based probe, 3-alkyne, which could facilitate either visualization or affinity enrichment through the click conjugation catalyzed by copper. As expected, 3-alkyne modified AKT1(E17K) in a dose-dependent manner, showing a more effective capacity for covalent modification at Lys17 than at Glu17. Given the absence of a selective AKT1(E17K) inhibitor, it remains unclear whether blocking the endogenous mutant AKT1 alone is sufficient to inhibit tumor growth. To address this, the authors tested LAPC4-CR tumor cells, and this cell line carries the heterozygous mutation site on E17K. Exposure to Compound 3 significantly reduced cell viability compared with ARQ092. Moreover, a negative control was set up using SkBr3 breast cancer cells, which do not carry the E17K mutation. Notably, the authors found that the tumor cells exhibited 50-fold lower sensitivity to Compound 3, and 3 times less sensitive to ARQ092 than LAPC4-CR prostate cancer cells. In summary, these results demonstrate that Compounds 1–3 have a superior ability to selectively target the mutant AKT1(E17K) over pan-AKT inhibitors.

To address the challenges of in vivo application, the authors modified the lysine-targeted salicylaldehyde inhibitors and developed Compound 4, which demonstrated improved pharmacokinetics and selectivity for in vivo use. In LAPC4-CR-bearing mice, a single application of Compound 4 effectively suppressed the AKT1 signaling pathway and tumor growth without significantly affecting blood glucose levels. As anticipated, Compound 4 also showed inhibitory effects in a breast cancer model with E17K-mutant. By contrast, the authors also found that a WT AKT1 breast cancer xenograft model displayed reduced sensitivity to compound 4, but showed higher sensitive to ARQ092 and capivasertib, both of them are regular pan-AKT inhibitors. These results demonstrate its potential as a safer and more effective treatment option (Fig. 1c).

Moreover, the authors resolved the crystal structure of AKT1(E17K) in the complex with the Compound 3, revealing that this lysine-targeted salicylaldehyde inhibitor interacts with Lys17 via the expected imine bond. Interestingly, the electron density map indicated that a tetravalent metal ion may coordinate with both the salicylaldehyde inhibitor and the activation loop of the kinase domain (Fig. 1b). Based on its distinct tetrahedral geometry and bond lengths, the authors identified Zn^2+^. They suggested that the Zn^2+^ ion might stabilize the salicylaldimine-Lys17 conjugate, although its precise biological role remains unclear. To investigate further, the authors performed differential scanning fluorimetry by adding Zn^2+^ to the Compound 3-AKT1(E17K) complex, observing a significant increase in thermal stabilization. Notably, this effect was abolished upon mutation of C296/310A, emphasizing the essential role of the Zn^2+^ chelate. Furthermore, cellular thermal shift assays confirmed that this Zn^2+^ chelate functioned similarly in living cells. Meanwhile, in order to validate the bound metal plainly, the authors tried to purify the complex from cells with the flag beads and analyzed the elutions by inductively coupled plasma mass spectrometry (ICP-MS) to quantify the most abundant biologically relevant transition metals. The authors found that only compound 3 could led to the selective enrichment of Zn^2+^ but not the cysteine double mutant. What’s more, the authors found that the Zn^2+^ was not enriched with FLAG-AKT1(E17K) purified form cells treated with DMSO instead of compound 3. The all above results indicate that the Zn^2+^ chelation plays a critical role in the AKT1(E17K) inhibition.

In conclusion, this study introduces a targeted strategy for inhibiting the AKT mutation. By designing highly selective inhibitors (Compounds 1–4), the authors addressed the limitations of existing therapies and paved the way for more effective treatments for AKT1 mutation-driven cancers. Notably, the authors also emphasized the promising in vivo targeting potential of these compounds, marking a significant advancement toward the efficient treatment of mutant AKT-driven solid tumors in clinical settings. Since the mutant AKT1 is implicated in the onset of a wide range of solid tumors, this research provides a new approach for mutant-selective AKT1-targeted therapies, with fewer side effects.

## Data Availability

Not Applicable.
